# Effects of field history on resistance to Bt maize by western corn rootworm, *Diabrotica virgifera virgifera* LeConte (Coleoptera: Chrysomelidae)

**DOI:** 10.1371/journal.pone.0200156

**Published:** 2018-07-03

**Authors:** Ram B. Shrestha, Mike W. Dunbar, Bryan W. French, Aaron J. Gassmann

**Affiliations:** 1 Department of Entomology, Iowa State University, Ames, Iowa, United States of America; 2 USDA-ARS, North Central Agricultural Research Laboratory, Brookings, South Dakota, United States of America; University of Illinois at Urbana-Champaign, UNITED STATES

## Abstract

Western corn rootworm, *Diabrotica virgifera virgifera* LeConte, has evolved resistance to transgenic maize, *Zea maize* L., that produces the insecticidal protein Cry3Bb1, which is derived from the bacterium *Bacillus thuringiensis*. We hypothesized that the level of Cry3Bb1 resistance in populations of western corn rootworm could be influenced by farming practices. To test this hypothesis, we evaluated the effect of field history on resistance to Cry3Bb1 maize by western corn rootworm. In 2013 and 2014, rootworm adults were collected from the four types of maize fields: 1) current problem fields, 2) past problem fields, 3) rotated maize fields, and 4) continuous maize fields. Those field populations along with seven Bt-susceptible control populations were tested for Cry3Bb1 resistance with both plant-based and diet-based bioassays. All field populations were resistant to Cry3Bb1 regardless of field history, however, some variation in the degree of resistance was found. For all categories of field populations, larval survivorship on Cry3Bb1 maize was significantly higher than control populations, and did not differ from survival on non-Bt maize. Evidence of resistance to Cry3Bb1 maize in plant-based bioassays was further supported by diet-based bioassays and we found a positive relationship between LC_50_ values from diet-based bioassays and the larval survivorship in plant-based bioassays. This study provides evidence of Cry3Bb1 resistance throughout the agricultural landscape studied, irrespective of the field history, and highlights the need for improved resistance management approaches, such as better use of integrated pest management to better delay pest resistance.

## Introduction

Western corn rootworm, *Diabrotica virgifera virgifera* LeConte (Coleoptera: Chrisomelidae), is among the most serious pests of maize in the United States [1, 2]. Larval western corn rootworm can cause severe injury to maize roots and reduce yield causing economic losses of greater than $1 billion annually from both management costs and yield loss [2–4]. Historically, management of western corn rootworm has included crop rotation and conventional insecticides [5, 6]. In 2003, genetically engineered maize producing the Cry3Bb1 toxin derived from the bacterium *Bacillus thuriengensis* (Bt) was released for management of corn rootworm [7]. Currently, Bt maize hybrids producing four crystline (Cry) toxins (i.e., Cry3Bb1, mCry3A, Cry34/35Ab1, and eCry3.1Ab) either singly, or as a pyramid of two toxins, are available for management of corn rootworm [8].

Bt crops can prevent yield loss from insect pests and reduce reliance on conventional insecticides [9–11]. However, planting Bt crops selects for Bt resistance, which can diminish the benefits of Bt crops [12, 13]. Field-evolved resistance can be defined as “a genetically based decrease in the susceptibility of a population to a toxin caused by exposure of the population to the toxin in the field” [14]. When field-evoled resistance reduces the efficiacy of Bt crop and has practical implication for pest management it can be defined as practicall ressistance [14]. To date, practical field-evolved resistance to Bt crops has been reported in one coleopteran pest, western corn rootworm, and six lepidopteran pests: corn earworm, *Helipcoverpa zea* (Boddie); maize stem borer, *Busseola fusca* (Fuller); sugercane borer, *Diatraea saccharalis* (Fabricius); western bean cutworm, *Striacosta albicosta* (Smith); fall armyworm, *Spodoptera frugiperda* (Smith); and pink bollworm, *Pectinophora gossypiella* (Saunders) [15,16]. In addition, cotton bollworm, *Helicoverpa armigera* (Hübner), and Asian corn borrer, *Ostrinia furnacalis* (Guenee), have showen reduced susceptibility to Bt crops, but the practical efficacy of these Bt crops has not been reduced to this point [16].

Beginning in 2009, field-evolved Bt resistance by western corn rootworm to Cry3Bb1 maize was found in northeastern Iowa [12]. Subsequently, resistance to Cry3Bb1 was detected in fields distributed throughout Iowa [13, 17, 18]. Populations of Cry3Bb1-resistant western corn rootworm also have been identified in Illinois, Nebraska and Minnesota [19–21]. Western corn rootworm have evolved resistance to all four currently available Bt toxins (Cry3Bb1, mCry3A, eCry3.1Ab, and Cry34/35Ab1) [13, 18, 21–23]. Furthermore, cross-resistance among three Cry toxins, Cry3Bb1, mCry3A and eCry3.1Ab, has been found for field populations of western corn rootworm [13, 18, 19, 21].

The large-scale adoption of Cry3Bb1 maize and the limited dispersal of rootworm adults has caused intense local selection in fields that are planted to Cry3Bb1 maize continuously [24, 25]. Past research has shown rapid evolution of Cry3Bb1 resistance by western corn rootworm in some fields, with resistance developing after three or more years of continuous cultivation of Cry3Bb1 maize, which translates to three generations for western corn rootworm [12, 13, 17]. The rate of evolution of resistance to a Bt crop depends on several factors such as the intensity of selection for Bt resistance, initial frequency of resistant alleles, degree of dominance for a resistance trait and whether or not fitness costs accompany Bt resistance [26–29].

All Bt maize hybrids developed for rootworm management, including Cry3Bb1 maize, do not produce a high-dose of Bt toxin (i.e., cause 99.99% mortality of Bt susceptible individuals) [8, 16]. This lack of high-dose means that some heterozygous resistant individuals can survive on Bt maize, which heightens the risk of pest populations developing resistance [30]. Additionally, the initial frequency for resistance alleles may have been as high as 0.1 which is ten times higher than originally expected [31]. In general, it appears that resistance to Cry3Bb1 maize by western corn rootworm is inherited as a non-recessive trait, which is consistent with a lack of a high-dose of Bt toxin, and has few accompanying fitness costs. All of these factors may have contributed to the development of resistance in the field [32–34].

Western corn rootworm larvae can survive to adulthood on only a limited number of plant species [35–37]. Therefore, rotation of non-host crops such as soybean could be an effective strategy to manage western corn rootworm [2, 38]. However, females of the rotation-resistant variant of western corn rootworm oviposit in crops other than maize, circumventing crop rotation as a management tool in some regions [6]. While the rotation-resistant variant is prevalent in Illinois and Indiana, it is rare or absent in other states, such as Iowa [39]. Crop rotation can drastically reduce the size of western corn rootworm populations, and remains a useful tool for managing populations of western corn rootworm, including those with Bt resistance. Crop rotation is a widely accepted strategy for management of corn rootworm, but the effect of crop rotation on Bt resistance has not been characterized. Additionally, selection pressure for Bt resistance in western corn rootworm can vary from field to field based on cropping history. From a resistance-management perspective, it is important to understand the effect of field history, including crop rotation, on the evolution of Bt resistance in the field.

We hypothesized that field history may affect the level of Bt resistance by western corn rootworm. To address this hypothesis, we evaluated the effect of the field history on resistance to Cry3Bb1 maize by western corn rootworm. Single-plant bioassays and diet-based bioassays were used to quantify resistance in western corn rootworm populations sampled from the four types of maize fields: 1) fields with a history of more than one node root injury to Cry3Bb1 or mCry3A maize, 2) maize fields with more than one node root injury to Cry3Bb1 or mCry3A at the time of sampling, 3) maize fields that had been rotated to an alternative crop two years previously, and 4) maize fields that had been planted to maize continuously for more than seven years. Knowledge generated from this study will be useful for developing better resistance management strategies for Bt crops targeting corn rootworm and other agricultural pests, and for increasing the effectiveness of current practices for managing Cry3Bb1-resistant populations of western corn rootworm.

## Methods

The effect of field history on Cry3Bb1 resistance by western corn rootworm was evaluated by sampling field populations of western corn rootworm adults in Iowa, USA and performing subsequent larval bioassays on their progeny in the laboratory. The field populations evaluated in this study were the same as those characterized in Dunbar et al. [40]. Collection of adult western corn rootworm occurred in the first three weeks of August in 2013 and 2014. Larval bioassays were conducted between July and October of 2014, for field populations sampled in 2013, and between March and July of 2015, for field populations sampled in 2014.

### Collection of field populations

**Fig 1:**
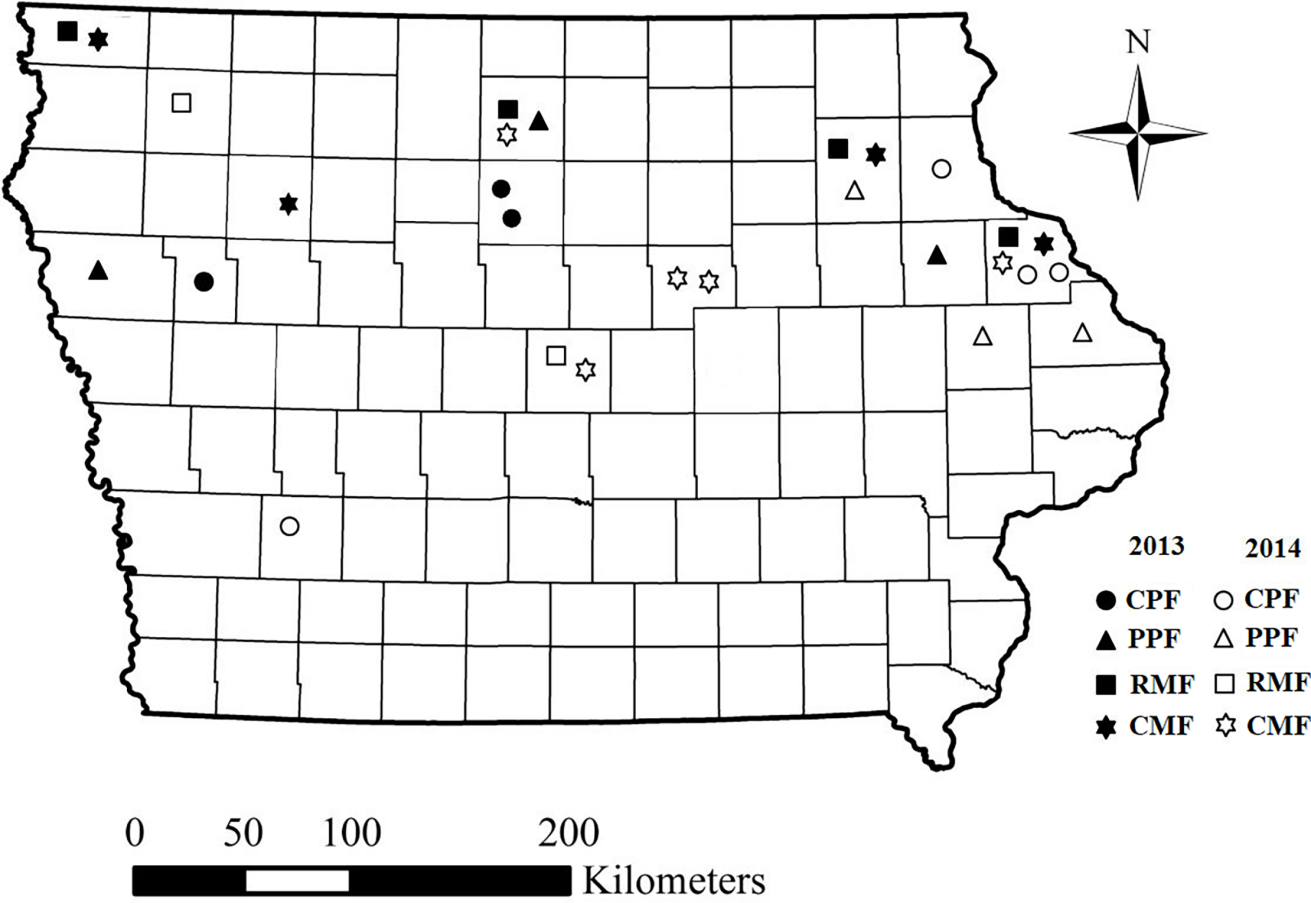
Location of western corn rootworm populations tested in plant-based bioassays. The map represents the state of Iowa, USA and individual sections of the map are counties in Iowa. Symbols represent individual fields that were sampled for adult western corn rootworm and subsequently tested in bioassays. Field locations are accurate to the level of an individual county. CPF = current problem field, PPF = past problem field, RMF = rotated maize field, and CMF = continuous maize field.

Western corn rootworm adults were collected from maize fields that were studied by Dunbar et al. [40]. Briefly, Dunbar et al. [40] studied a total of 47 maize fields in Iowa that were selected with the help of regional agronomists and local cooperators. Twenty maize fields were visited in 2013 and 27 maize fields were visited in 2014. For all fields, Dunbar et al. [40] reported abundance of adult rootworm *Diabrotica* spp., larval injury to maize roots, and cropping history. The field populations were collected from the maize fields in 15 counties in the northern Iowa, and one county in southwest Iowa (Fig 1). In all cases, the owner of the land gave permission to conduct this study in their field.

Maize fields were categorized based on either root injury to Bt maize or how rootworm were managed. Dunbar et al. (2016) quantified the level of root injury in maize fields by sampling 12 roots from each field. These roots were then rated for rootworm feeding injury using the 0 to 3 node injury scale of Oleson et al. [41]. Each field belonged to one of four categories: 1) current problem fields (CPF), maize fields with >1 node root injury to Cry3 maize (i.e., either Cry3Bb1 maize or mCry3A maize) at the time of sampling, 2) past problem fields (PPF), maize fields with a history of >1 node root injury to Cry3 maize, 3) rotated maize fields (RMF), second-year maize fields that were planted to maize during the previous growing season but an alternative crop two years previous, 4) continuous maize fields (CMF), fields that were planted to maize continuously for 7 or more years.

Each of the four categories of field populations had a combination of attributes that were unique from the other categories, and thus, were mutually exclusive. Specifically, continuous maize fields had both continuous maize cultivation for at least 7 years plus an absence of greater than expected injury to Bt maize in the past. Rotated maize fields had been rotated out of maize production two years prior to sampling and did have a history of greater than expected injury to Bt maize. Past problem fields had a history of greater than expected injury to Bt maize one to five years prior to the growing season in which they were sampled for this study and current problem fields experienced greater than expected injury to Bt maize in the season they were sampled. While field types may share some attributes, for example both continuous maize fields and past problem fields, may have been planted to maize for several consecutive years, they also have other attributes that are not shared (i.e., the presence versus absence of high levels of feeding injury to Bt maize). Thus, each category is unique and mutually exclusive from other categories. Of the 47 fields studied by Dunbar et al. [40], western corn rootworm adults were sufficiently abundant to obtain samples from 31 fields: CPF (N = 9), PPF (N = 6), CMF (N = 9), and RMF (N = 7). Adult western corn rootworm (N = 49 to 440; 230.7 ± 104.7 Mean ± SD) were collected from each of these 31 fields and brought to the laboratory. Each population was held in a separate cage (18 cm × 18 cm × 18 cm; L × W × H) (Megaview Science, Taiwan) in an incubator (25°C; 16/8 L/D) to collect eggs. Insects were fed a complete adult diet (western corn rootworm adult diet, product # AG-F9768B-M, Frontier Agricultural Sciences, Newark, DE) and leaf tissue of maize. A 1.5% agar solid was provided as a source of water, and sieved soil (< 180 μm) held in a Petri dish (diameter = 10 cm) served as an oviposition substrate. Eggs were collected from each population (N = 1,000 to 35,000; 13,329 ± 9,403 Mean ± SD) and stored at 6°C for at least 5 months to break diapause. However, we could not evaluate a population from one of the rotated maize fields because the number of eggs collected (less than 1,500 eggs) was not sufficient to conduct either plant-based or diet-based bioassays.

### Bioassays

The level of Cry3Bb1 resistance was determined using both plant-based bioassays and diet-based bioassays. We obtained a sufficient number of eggs (i.e., > 1500 per population) to conduct plant-based bioassays for 30 populations. Additionally, for 20 field populations, we obtained sufficient eggs (>7,000 eggs per population) to carry out both plant-based and diet-based bioassays. More eggs were needed for diet-based bioassays because these assays require 72 larvae per bioassay plate, versus 12 larvae per plant in plant-based bioassays.

### Plant-based bioassay

Plant-based bioassays were conducted following Gassmann et al. [13]. Briefly, maize plants were grown in a greenhouse, with individual plants held in 1 L plastic containers with potting medium. Plants were grown for 4 to 5 weeks until they reached the V5 to V6 growth stage (i.e., 5 to 6 leaf stage [42]). Plants were trimmed to a height of 20 cm and 12 neonate larvae (<24 h old) were placed on roots at the base of the plant. Plants with larvae were held in a growth chamber for 17 days (24°C; 16/8 L/D; 60% RH). After 17 days, live larvae were extracted from roots and potting medium into vials with 85% ethanol using a Berlese funnel, and larvae then counted under a stereo microscope (MZ6, Leica, Microsystems, Wetzlar, Germany). For all larvae collected from plant-based bioassays, larval instar was determined based on head-capsule width following Hammack et al. [43].

A total of 30 field populations and 7 control populations were tested using plant-based bioassays. The number of populations, per field type, evaluated with the plant-based bioassays was: CPF = 9, PPF = 6, CMF = 9, and RMF = 6, and these populations represent samples from across the state of Iowa (Fig 1). Sixteen field populations were tested in 2014 and fourteen field population were tested in 2015. In addition to field populations, we also tested seven Bt-susceptible control populations. Control populations were originally collected from Moody Co., South Dakota in 1987; Phelps Co., Nebraska in 1995; Potter Co., South Dakota in 1995; York Co., Nebraska in 1996; Butler Co., Nebraska in 1999; Finney Co., Kansas in 2000; and Centre Co., Pennsylvania in 2000. Control populations (CTRL) were provided as eggs in the diapause stage from the United States Department of Agriculture, Agricultural Research Service, North Central Agricultural Research Laboratory in Brookings, South Dakota. All control populations were brought into laboratory culture before 2003, which is the year Bt maize was registered for management of corn rootworm. Thus, these populations represent Bt-susceptible control populations because they never experienced selection for Bt resistance in the field or laboratory. At least one control population and one to three field populations were tested every week for 7 weeks in 2014 and 9 weeks in 2015. During the entire experiment, control populations were tested a total of eighteen times, with each control population evaluated the following number of times: Moody = 4, Finney = 4, Phelps = 3, Centre = 3, Butler = 2, Potter = 1, and York = 1. Field populations were tested only once. Each population was tested against Cry3Bb1 maize (hybrid DKC 6169; DeKalb Brand; Monsanto Co., St. Louis, Missouri) and non-Bt near isoline to Cry3Bb1 maize (hybrid DKC 6172). For each combination of population by hybrid, 10 maize plants were used in 2014 and 12 plants in 2015, each time a population was tested.

### Diet-based bioassay

Diet-based bioassays using Cry3Bb1 toxin were conducted for a total of 27 western corn rootworm populations (20 field populations and seven control populations). Both field populations and control populations tested in diet-based bioassays were the same as those tested in plant-based bioassays. Sample sizes were CPF = 5, PPF = 5, CMF = 5, RMF = 5, with 16 populations tested in 2014 and four in 2015. Six control populations were tested in 2014 and five in 2015. Diet bioassays were run from July to October in 2014 and from March to May in 2015. At least one control population, along with one to four field populations, were tested per week, and control populations were alternated weekly.

Diet-based bioassays were conducted following Siegfried et al. [44]. Briefly, western corn rootworm eggs that were ready to hatch in 1 to 2 days were cleaned with salt-water flotation following Shaw et al. [45]. Eggs were then surface sterilized by soaking in a 2% bleach solution for 3 min followed by rinsing with distilled water. Eggs were further surface sterilized by soaking in 800 ppm Roccal-D Plus solution (Pfizer, Inc. New York, NY) for 3 min and then rinsed with distilled water. Eggs were then placed on filter paper (Pure Brew, 7.6 cm base, coffee filter, Rochline, Sheboygan, Wisconsin) that had first been sterilized by soaking in 800 ppm Roccal-D solution and dried in a sterile hood (PCR Workstation, Fisher Scientific Company, Nazareth, Pennsylvania). Cleaned and sterilized eggs on sterilized filter paper were placed on a 2% agar solid (S70213A, Fisher Scientific Company) in a 0.5 L container (DeliPRO, TD40016, TRiPAK Industrial USA, LLC, White Plains, New York). Containers with eggs were placed in a incubator (26.7°C, 0/24 L/D) until hatching. Diet plates, Cry3Bb1 toxin, and buffer were supplied by Monsanto Co. (St. Louis, Missouri). Serial dilutions of Cry3Bb1 toxin in buffer were made to produce six concentrations (341.6, 170.8, 85.4, 42.7, 21.4, and 0 μg/cm^2^ of diet surface). Buffer without toxin was used as the control. Buffer solution (20 μL) with the appropriate amount of Cry3Bb1 was added to the surface of the diet in each well. Twelve wells in each diet plate (Falcon™ Polystyrene Microplates, [353916, flat bottom, 96 well, 0.32 cm^2^ area] Corning, Inc., Corning, New York) were coated with each concentration of toxin and allowed to dry for 2 to 3 hours in a sterile hood. Once dry, a single neonate larva was placed in each well and the plate was covered with adhesive plate seal (part number AB0580, Fisher Scientific Company). Two small holes were made over each well to allow ventilation. Bioassay plates with larvae were placed in an incubator (26.7 °C, 67% RH, 0/24 L/D) for 5 days. After 5 days, plates were inspected under a dissecting microscope and larval mortality recorded.

### Data analysis

For plant-based bioassays, proportion larval survival was calculated as the number of larvae recovered after 17 days divided by the number of neonates initially placed in the bioassay container (i.e., 12). We set a threshold of at least 14% average survival on the non-Bt isoline for a population to be included in data analysis. With the exception of two of nine current problem fields, all other field populations tested met this threshold. For control populations, four of the 18 sets of assays were below this threshold. For the remaining 14 sets of assays, data from within an individual control population were combined for analysis. After excluding the sets of bioassays that did not meet the threshold, our final sample size was 28 field populations and seven control populations. Two complementary analyses were conducted for data from plant-based bioassays. One method tested for resistance among each category of field type (e.g., past problem fields or rotated maize fields etc.), and the other method tested for resistance in the western corn rootworm populations from each of the individual fields.

To test for resistance among each category of field population, data from plant-based bioassays (proportion larval survivorship and proportion of third instar larvae) were analyzed with a mixed-model analysis of variance (ANOVA) (PROC GLIMMIX) in SAS 9.4 [46]. The Laplace method of maximum likelihood estimation (binomial distribution and logit link function) was used to fit the model. Fixed effects included rootworm population type (CPF, PPF, CMF, RMF, and control), maize hybrid (Cry3Bb1 maize vs. non-Bt near isoline), and their interaction. Random effects included population nested within population type and the interaction between maize hybrid and population nested within population type. Because of the significant interaction between maize hybrid and population type, pairwise comparisons were made among population types within each maize hybrid (20 comparisons in total) and between maize hybrids within each population type (5 comparisons in total). Treatment means were compared with LSMEANS procedure using a significance level of *P* < 0.002 based on Bonferroni correction for 25 pairwise comparisons [47]. The treatment means, and standard errors were estimated with ilink function in PROC GLIMMIX.

To test for resistance in western corn rootworm populations from each individual field, corrected larval survivorship for each population was calculated as the complement of correct mortality based on the formula of Abbott [48], with survival on Cry3Bb1 maize in each bioassay container divided by average survival on non-Bt maize. To test whether a field population was resistant to Cry3Bb1 maize, we compared corrected survival for that field population against average corrected survival of the control population with a one-tailed t-test (PROC TTEST in SAS 9.4). The null hypothesis was that correct survival did not differ between a field population and the control populations (i.e., field population was susceptible to Cry3Bb1 maize), and the alternative hypothesis was that corrected survival was significantly greater for a field population compared to control populations (i.e., the field population was resistant to Cry3Bb1 maize).

To determine whether there was an effect of Bt maize on an individual population, we compared both survival and development (i.e., proportion third instar) on Cry3Bb1 maize to non-Bt maize for each field population with a one-tailed t-test (PROC TTEST). In both cases, the null hypothesis was that survival/development was equal between Cry3Bb1 and non-Bt maize and the alternative hypothesis was that survival/development was less on Cry3Bb1 maize compared to non-Bt maize. For all t-tests, a folded-F test was used to determine the equity of the variance, with pooled variance used for cases with equal variance, whereas a Satterthwaite correction was applied when the variance was unequal. The statistical power of each t-test was determined by conducting a power analysis (PROC POWER) using two sample means, standard deviations, normal distribution, and one-sided test option. In addition, Test = diff_satt option was used when variances were heterogeneous, however Test = diff option and pooled standard deviation was used when variances were homogeneous. Finally, to test whether survival or development differed between field populations and control populations on non-Bt maize, which might indicate an overall difference in vigor or fitness between field and control populations in plant-based bioassays, two-tailed t-tests were conducted.

For diet-based bioassays, an individual replicate (i.e., a single 96 well plate) was only included in the data analysis if at least 67% survival was observed in control wells. During the entire experiment, a total of 344 diet-plate assays were run (251 plates in 2014 and 93 plates in 2015). However, data from 43% of the plates (N = 149) were discarded due to high larval mortality in control wells (i.e., mortality > 33%). A total of twelve populations (eight field populations and four control populations) had four or more diet plates with sufficiently low control mortality and were used to determine LC_50_ values and associated statistics. Larval mortality data from the diet bioassays were subjected to probit analysis (PROC PROBIT). LC_50_ values (μg/cm^2^ of diet surface) and 95% fiducial limits were calculated with INVERSECL function, and goodness of fit was tested based on Pearson’s Chi-square.

The relationship between survival on Cry3Bb1 maize in plant-based bioassays and LC_50_ values in diet-based bioassays was determined by correlation analysis (PROC CORR). The analysis used LC_50_ values from diet-based bioassays and corrected larval survivorship from plant-based bioassays for eight field populations and four control populations.

## Results

### Larval survivorship and development in plant-based bioassays

**Fig 2:**
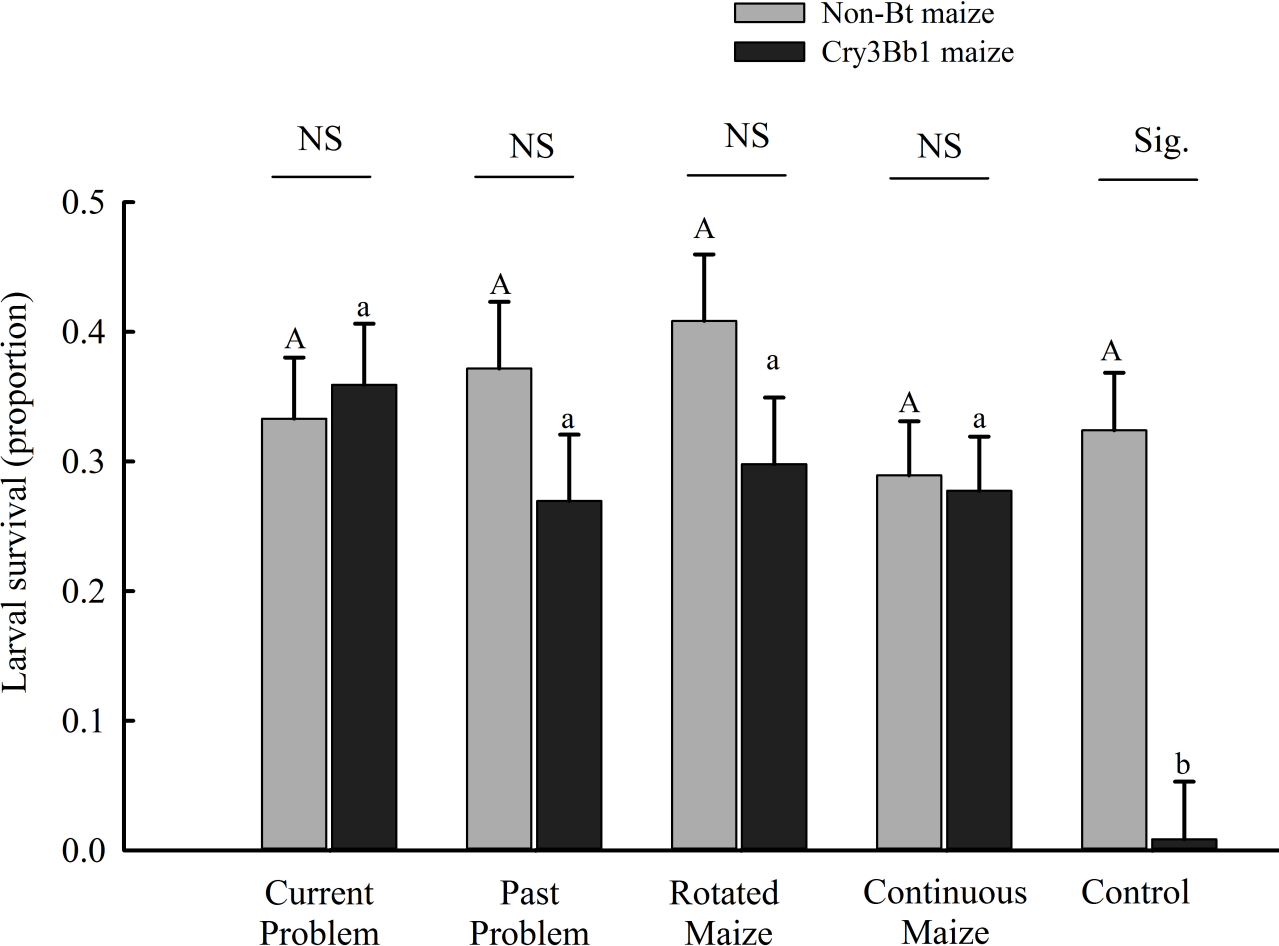
Survival of western corn rootworm larvae on Cry3Bb1 maize and non-Bt maize in plant-based bioassays. Bar heights are sample means and error bars are the standard error of the mean. Uppercase letters indicate significant differences between population classes on non-Bt maize and lowercase letters indicate significant differences between population classes on Cry3Bb1 maize. Differences between Cry3Bb1 maize and non-Bt maize within a population class are indicated by either NS for a non-significant difference or Sig., which stands for a significant difference.

Analysis of larval survivorship by population type revealed a significant interaction between population type and maize hybrid (Table 1). Larval survivorship on Cry3Bb1 maize was similar among the four classes of field populations (CPF, PPF, CMF, and RMF) and was significantly higher than control populations (Fig 2). By contrast, larval survival on non-Bt maize was similar, and not significantly different, among all population types. No significant difference between survival on Cry3Bb1 maize and non-Bt maize was observed among the four classes on field populations, but by contrast, survival of control populations was significantly lower on Cry3Bb1 maize compared to non-Bt maize (Fig 2).

**Table 1:** Analysis of variance for data from plant-based bioassays.

Fixed effect	Proportion survival of larvae			Proportion of third-instar larvae ^1^		
	*df*	*F*	*P*	*df*	*F*	*P*
Population type	4, 30	16.06	<0.001	4, 27	2.84	0.044
Maize hybrid	1, 30	96.86	<0.001	1, 27	32.62	<0.001
Population type × Maize hybrid	4, 30	39.23	<0.001	4, 27	3.92	0.012

^1^The number of third instar larvae divided by the total number of larvae recovered at the end of the assay.

When data were analyzed by population type, there also was a significant interaction between population type and maize hybrid for the proportion of third instar larvae (Table 1). All population types displayed similar development on non-Bt maize (Table 2). The proportion of third instar larvae on Cry3Bb1 maize and non-Bt maize was similar for current problem fields and continuous maize fields (Table 2). However, for past problem fields, rotated maize fields and control populations, the proportion of third instar larvae, (i.e., the number of third instar larvae divided by the total number of larvae that survived) on Cry3Bb1 maize was significantly lower than on non-Bt maize, indicating a developmental delay on Cry3Bb1 maize (Table 2). There was a higher level of adaptation to Cry3Bb1 maize in current problem fields and continuous maize fields than in rotated fields and past problem fields.

**Table 2:** Proportion of third instar larvae on Cry3Bb1 maize and non-Bt maize^1^.

Population type	Non-Bt Maize	Cry3Bb1 Maize
Current problem fields	0.61±0.04 a A	0.53±0.05 a A
Past problem fields	0.65±0.05 a A	0.45±0.05 b AB
Rotated maize fields	0.60±0.05 a A	0.42±0.05 b AB
Continuous maize fields	0.49±0.04 a A	0.43±0.04 a AB
Control	0.60±0.04 a A	0.09±0.06 b B

^1^Means followed by different lowercase letters are significantly different between hybrids within a population type and means followed by different uppercase letters differ between population types within a maize hybrid.

When corrected survival of each field population was tested against average corrected survival for control populations, all individual field populations had significantly greater corrected survival than the control populations (Table 3). When larval survivorship and developmental rate for each rootworm population were compared between Cry3Bb1 vs. non-Bt maize, 75% of the field populations (21 out of 28 field populations) did not differ for either of these factors on Cry3Bb1 maize vs. non-Bt maize (Table 3). The other seven field populations showed either only delayed development (N = 2), only reduced survivorship (N = 2) or both delayed development and reduced survival (N = 3) on Cry3Bb1 maize compared to non-Bt maize. The distribution of populations where an effect of Cry3Bb1 on either larval survival or larval development or both was detected was: continuous maize fields (1 of 9), current problem fields (2 of 7), past problem field (3 of 6) and rotated maize fields (1 of 6). However, the power of these individuals tests was variable, and in some cases low (1 – β ranged from 0.06 to 1.00) suggesting that there were likely additional differences that we lacked the statistical power to detect.

**Table 3:** Larval survivorship of western corn rootworm in plant-based bioassays.

Populations	Uncorrected survival			Corrected survival	Proportion of third instar larvae		
	Non-Bt^1^	Bt	Non-Bt> Bt^2^	Bt^3^	Non-Bt^1^	Bt	Non-Bt > Bt^2^
Continuous maize							
CMF1	0.27±0.06 b	0.18±0.04	NS	0.66±0.16 A	0.71±0.12 b	0.61±0.13	NS
CMF2	0.20±0.07 b	0.10±0.02	NS	0.52±0.12 A	0.33±0.09 a	0.26±0.12	NS
CMF3	0.39±0.06 b	0.42±0.07	NS	1.08±0.18 A	0.51±0.11 b	0.56±0.09	NS
CMF4	0.50±0.07 a	0.46±0.03	NS	0.92±0.07 A	0.52±0.08 b	0.51±0.08	NS
CMF5	0.32±0.07 b	0.19±0.05	NS	0.61±0.17 A	0.57±0.10 b	0.29±0.07	S
CMF6	0.28±0.07 b	0.35±0.05	NS	1.23±0.16 A	0.53±0.09 b	0.52±0.10	NS
CMF7	0.21±0.06 b	0.18±0.05	NS	0.88±0.26 A	0.49±0.14 b	0.57±0.14	NS
CMF8	0.21±0.05 b	0.33±0.06	NS	1.56±0.31 A	0.36±0.13 a	0.33±0.09	NS
CMF9	0.21±0.05 b	0.29±0.04	NS	1.40±0.18 A	0.34±0.10 a	0.14±0.07	NS
Current problem							
CPF1	0.52±0.06 a	0.49±0.06	NS	0.95±0.11 A	0.36±0.07 a	0.26±0.04	NS
CPF2	0.30±0.08 b	0.47±0.07	NS	1.59±0.22 A	0.75±0.10 b	0.68±0.08	NS
CPF3	0.25±0.06 b	0.44±0.07	NS	1.75±0.30 A	0.39±0.11 b	0.61±0.11	NS
CPF4	0.26±0.06 b	0.39±0.08	NS	1.50±0.31 A	0.39±0.14 b	0.68±0.11	NS
CPF5	0.34±0.07 b	0.12±0.05	S	0.34±0.13 A	0.69±0.09 b	0.34±0.17	S
CPF6	0.23±0.06 b	0.29±0.04	NS	1.30±0.19 A	0.52±0.11 b	0.24±0.07	S
CPF7	0.45±0.07 b	0.30±0.08	NS	0.67±0.18 A	0.65±0.09 b	0.47±0.13	NS
Past problem							
PPF1	0.16±0.04 a	0.16±0.04	NS	0.99±0.25 A	0.43±0.12 b	0.41±0.12	NS
PPF2	0.45±0.08 b	0.27±0.06	S	0.59±0.12 A	0.82±0.10 a	0.37±0.14	S
PPF3	0.54±0.08 a	0.35±0.07	S	0.66±0.12 A	0.63±0.04 b	0.54±0.09	NS
PPF4	0.69±0.06 a	0.45±0.06	S	0.66±0.09 A	0.53±0.11 b	0.45±0.06	NS
PPF5	0.19±0.06 b	0.27±0.06	NS	1.38±0.29 A	0.63±0.14 b	0.44±0.12	NS
PPF6	0.22±0.07 b	0.11±0.03	NS	0.50±0.14 A	0.68±0.14 b	0.45±0.16	NS
Rotated maize							
RMF1	0.33±0.09 b	0.21±0.05	NS	0.64±0.16 A	0.47±0.10 b	0.35±0.12	NS
RMF2	0.53±0.10 a	0.41±0.06	NS	0.77±0.11 A	0.63±0.07 b	0.56±0.06	NS
RMF3	0.48±0.08 b	0.39±0.05	NS	0.81±0.11 A	0.64±0.09 b	0.44±0.07	NS
RMF4	0.56±0.06 a	0.32±0.05	S	0.57±0.09 A	0.58±0.07 b	0.33±0.07	S
RMF5	0.24±0.06 b	0.14±0.04	NS	0.58±0.18 A	0.32±0.10 a	0.19±0.09	NS
RMF6	0.31±0.09 b	0.33±0.07	NS	1.05±0.24 A	0.71±0.10 b	0.64±0.10	NS
Control strains							
CTRL1	0.51±0.04	0.02±0.01	NA	0.04±0.02	0.57±0.05	0.00±0.00	NA
CTRL2	0.45±0.06	0.02±0.01	NA	0.05±0.02	0.69±0.06	0.17±0.17	NA
CTRL3	0.25±0.04	0.00±0.00	NA	0.00±0.00	0.51±0.07	.	NA
CTRL4	0.32±0.06	0.00±0.00	NA	0.00±0.00	0.44±0.12	.	NA
CTRL5	0.25±0.04	0.02±0.01	NA	0.07±0.03	0.63±0.07	0.20±0.20	NA
CTRL6	0.15±0.04	0.00±0.00	NA	0.00±0.00	0.52±0.11	.	NA
CTRL7	0.32±0.05	0.01±0.00	NA	0.02±0.01	0.66±0.05	0.00±0.00	NA
AVG CTRL	0.34±0.02 b	0.01±0.00	NA	0.03±0.01 B	0.59±0.03 b	0.11±0.07	NA

^1^ Test for difference between average survival and development of control populations versus individual field population on non-Bt maize. Those field populations followed by an “a” differ significantly from the average value for the control populations, while means followed by “b” do not differ from controls.

^2^ S indicates proportion survival or proportion third instar is significantly lower on Bt maize compared to non-Bt maize for an individual field population; NS indicates survival or proportion third instar larvae was not significantly different between Bt maize and non-Bt maize for an individual field population; and NA indicates that a test was not applicable because data were for a control population.

^3^ Means of a field population followed by upper case letter “A” are significantly greater than average corrected survival for control populations.

When analyzed by individual population, 21 out of 28 field populations (75%) did not differ from control populations for survival on non-Bt maize, six field populaitons had significantly greater survival than control populations and one field populations had significantly lower survival (Table 3). Based on a sign test, the observation that six populations displaying greater survival and one population displaying lower survival does differ from the null expectation that an equal number of populations should display significantly lower vs. higher survival (P = 0.13) [38]. For larval development, 22 out of 28 field populations (79%) did not differ from control populations, one field population had significantly faster development and five field populations had significantly slower development (Table 3). Based on a sign test, the observation that five populations displayed slower development and one population displayed faster development does differ from the null expectation that an equal number of populations should display significantly faster vs. slower development (P = 0.22).

### Larval mortality in the diet-based bioassay and relationship with plant-based bioassays

**Fig 3:**
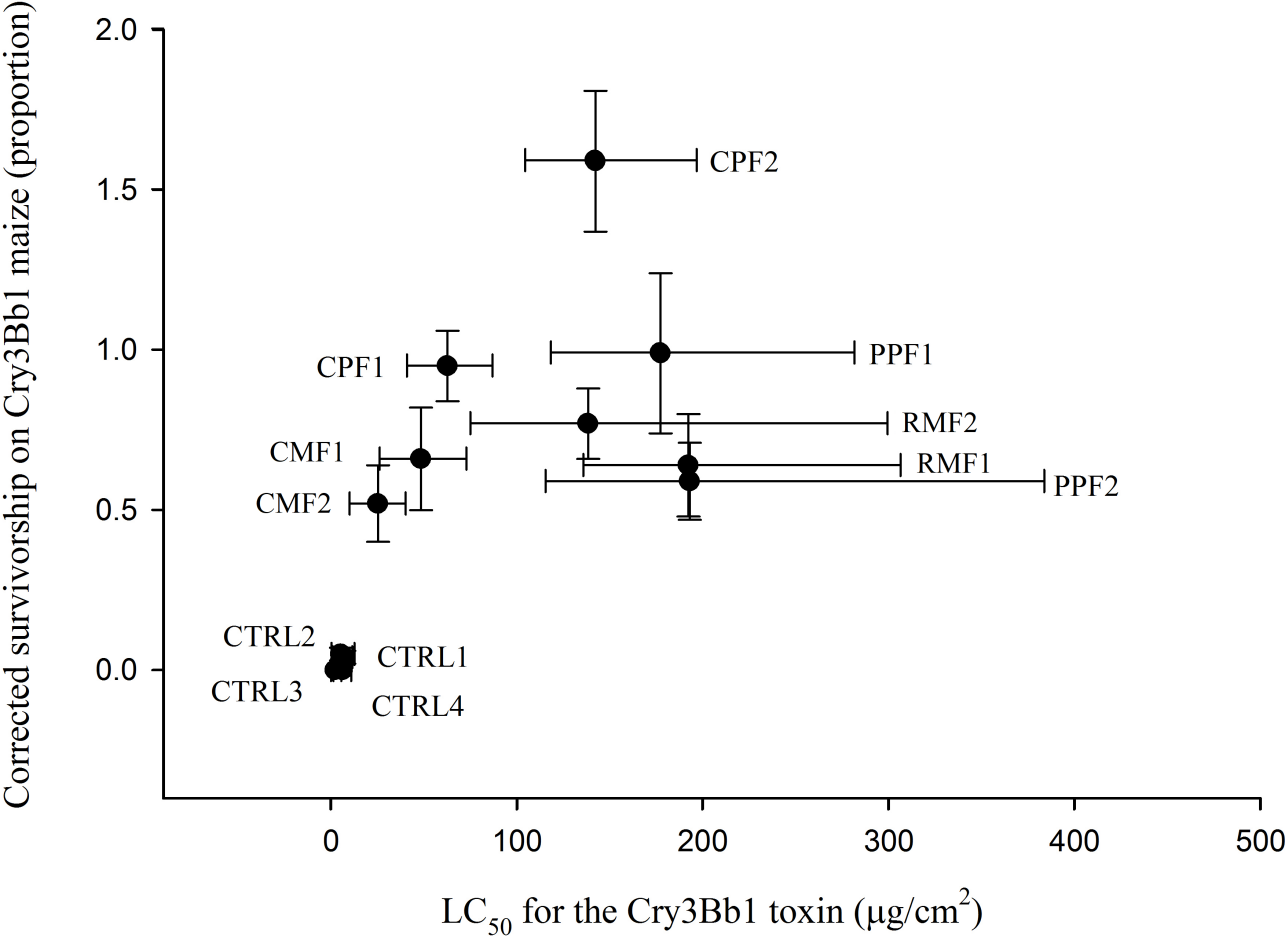
Relations between diet-based and plant-based bioassays for western corn rootworm. Horizontal lines represent 95% fiducial limits on LC_50_ values and vertical lines represent the standard error of mean for corrected larval survivorship on Cry3Bb1 maize. Individual points represent samples means. Lables by individual data points represent the follwing: CPF = current problem field, PPF = past problem field, RMF = rotated maize field, CMF = continuous maize field, and CTRL = control population.

For control populations, LC_50_ values for Cry3Bb1 protein ranged from 4.0 to 9.3 μg /cm^2^_,_ whereas field populations ranged from 25.3 to 192.3 μg /cm^2^ (Table 4)_._ Except for one continuous maize fields (CMF2), all field populations of western corn rootworm had significantly greater LC_50_ values than control populations, as evidenced by non-overlapping fiducial limits (Table 4). Additionally, the LC_50_ values for the two populations from continuous maize fields were significantly lower than populations from past problem fields and rotated maize fields (Table 4). Correlation analysis revealed a significant positive relationship between corrected survival in plant-based bioassays and LC_50_ values in diet-based bioassays (N = 12, *r* = 0.66, and *P* = 0.019) (Fig 3).

**Table 4:** Pobit analysis of larval mortality in diet-based bioassays.

Pop.^1^	Plate (n)^2^	Slope ± SE	LC_50_ (μg/cm^2^)	Lower FL^3^	Upper FL^3^	*df*	*X*^2^	P	RR^4^
CMF1	8	1.56 ± 0.28	48.08	26.57	72.55	3	5.18	0.16	6.9
CMF2	4	2.50 ± 0.83	25.29	10.36	40.32	3	7.03	0.07	3.6
CPF1	9	1.80 ± 0.27	62.12	40.88	86.52	3	3.25	0.36	9.0
CPF2	10	1.84 ± 0.26	141.55	104.16	196.21	3	4.34	0.23	20.4
PPF1	12	1.51 ± 0.26	177.31	118.37	280.40	3	0.44	0.93	25.6
PPF2	7	1.45 ± 0.32	192.25	115.00	381.25	3	3.28	0.35	27.7
RMF1	9	1.53 ± 0.25	192.29	136.39	304.04	3	1.12	0.77	27.7
RMF2	4	1.41 ± 0.38	137.99	74.49	299.76	3	0.98	0.81	19.9
CTRL1	14	6.17 ± 1.81	9.27	2.36	13.77	3	0.78	0.85	NA
CTRL2	7	1.86 ± 0.48	6.16	0.63	13.98	3	1.92	0.59	NA
CTRL3	17	3.26 ± 0.81	4.04	0.53	8.41	3	2.41	0.49	NA
CTRL4	11	4.30 ± 1.01	8.25	2.65	12.94	3	1.82	0.61	NA

^1^ CPF = Current problem field, PPF = Past problem field, RMF = Rotated maize field, CMF = Continue maize field, and CTRL = Control population (see Methods for additional descriptions of field types)

^2^ n = number of diet plates used in analysis

^3^ FL = 95% fiduciary limits

^4^ RR = resistance ratios were calculated by dividing LC_50_ value of a field population by the average LC_50_ values of control populations. NA = not applicable.

## Discussion

In this study field populations were sampled from a broad geographic range within Iowa, USA (Fig 1). Plant-based bioassays detected resistance to Cry3Bb1 maize for all field populations of western corn rootworm tested in this study (Fig 2, Table 3). Both corrected and uncorrected larval survival on Cry3Bb1 maize for field populations were higher than control populations, suggesting the presence of some level of Cry3Bb1 resistance in field populations. Diet-based bioassays conducted on a subset of these populations further supported the presence of resistance, with significantly higher LC_50_ values for Cry3Bb1 found for all but one field population compared to control populations (Table 4). Resistance to Cry3Bb1 was found irrespective of field history, although some variation among populations was detected in larval survivorship and developmental rate in plant-based bioassays and LC_50_ values in diet-based bioassays (Tables 3 and 4). In particular, western corn rootworm populations appeared to display greater adaptation to Cry3Bb1 maize in current problem fields and continuous maize fields, with no overall difference in larval survival or development detected for either of these classes of field populations (Fig 1). Both diet-based and plant-based assays detected Cry3Bb1 resistance, and there was a general positive relationship between survival on Cry3Bb1 maize in plant-based bioassays and LC_50_ values in diet-based bioassays (Fig 3).

The first case of field-evolved resistance to Cry3Bb1 maize was reported from four fields in northeast Iowa in 2009 by Gassmann et al. [12]. Subsequently, bioassays found increased survival on Cry3Bb1 maize over time for western corn rootworm sampled from fields with greater than one node of root injury to Cry3Bb1 maize [13]. The populations studied here were sampled in 2014 and 2015, which means that, after 5 to 6 years from the first reported cases of Cry3Bb1 resistance, Cry3Bb1 resistant populations were found widely distributed across Iowa (Fig 1). Western corn rootworm tends to exhibit limited dispersal [25], suggesting that localized selection was likely an important factor contributing to the resistance detected in this study. Consistent with this hypothesis, Dunbar et al. [40] observed that all field classes evaluated in this study typically had been planted to Cry3Bb1 maize in the past. The wide-spread distribution of Cry3Bb1-resistant western corn rootworm in Iowa maize fields indicates that the current strategy of planting refuge along with Bt maize may not be sufficient to prevent field-evolved Bt resistance by western corn rootworm.

When data were analyzed by field type (Fig 2 and Table 2), adaptation to Cry3Bb1 maize appeared to be greater in current problem fields and continuous maize fields than in rotated maize fields and past problem fields. This is based on the observation that, although all classes of field populations displayed similar survival on Cry3Bb1 maize and non-Bt maize (Fig 2), larval development on Cry3Bb1 maize was delayed compared to non-Bt maize for rotated maize fields and past problem fields (Table 2). The lesser degree of adaption in the past problem fields and rotated maize fields suggests that tactics adopted by farmers may have helped to delay or remediate Cry3Bb1 resistance. In the case of rotated maize fields, crop rotation may have promoted colonization of western corn rootworm from multiple fields, thus diminishing effects of local selection for Cry3Bb1 resistance. Because western corn rootworm larvae can survive only on roots of maize and few other grass hosts [35, 36], crop rotation can act to elminate the rootworm population within a field, with subsequent colonization from other fields then needed to re-establish the population. Crop rotation is applied only to a few fields at a time. Therefore, it is very likely that there will be Cry3Bb1 maize fields in the surrounding of recently rotated field. Cry3Bb1-resistant adults produced in the surrounding fields can easily fly into nearby newly rotated field and re-colonize. In the case of past problem fields, farmers appear to have altered their cropping practices after experiencing greater than expected injury to Cry3 maize, planting maize pyramided with Cry3Bb1 and Cry34/35Ab1, and growing less maize with Cry3Bb1 alone [40]. Shrestha et al. [49] found that use of Cry34/35Ab1 maize reduced emergence of adults that were resistant to Cry3Bb1. This decrease in emergence may have facilitated the subsequent dilution of resistance alleles by adult rootworm from neighboring fields, or non-Bt refuge plants, thus increasing the heterozygosity of resistance and possibly reducing the degree of adaption to Cry3Bb1. This hypothesis is consistent with the results of Deitloff et al. [50], in which laboratory selection experiments were conducted and it was found that introducing Bt susceptible rootworm delayed adaption to Bt maize. However, it remains an open question how biologically meaningful the delayed development observed for rootworm populations from rotated fields and past problem fields would be for either pest survival or injury to maize in the field.

Fitness costs associated with Bt resistance can reduce the level of resistance by removing the alleles for Bt resistance from a population once selection is removed [29]. In the absence of fitness costs, resistance can persist even though a population is no longer experiencing selection. Past research has found that fitness costs of Cry3Bb1 resistance in western corn rootworm tend to be minimal or absent altogether [32–34, 51–53]. Additionally, Cry3Bb1 resistance can persist up to six generation in the absence of selection [54]. If present, fitness costs could have contributed to the incomplete resistance observed in past problem fields compared to current problem fields (Table 2). However, that is likely not the case here because, following greater than expected injury to Cry3Bb1 maize in past problem fields, farmers predominantly planted maize pyramided with Cry34/35Ab1 and Cry3Bb1 [40], and this would have maintained selection for Cry3Bb1 resistance. More likely, the reduced emergence of Cry3Bb1-resistant individuals from pyramided maize combined with dilution of resistant genotypes by dispersal from nieghboring fields or non-Bt refuge plants led to the decreased degree of adaptation in past problem fields compared to current problem fields or continuous maize fields.

In general, western corn rootworm from continuous maize fields showed evidence of adaptation that was similar to current problem fields and greater than rotated fields or past problem fields, with rootworm larvae displaying an absence of a developmental delay on Cry3Bb1 maize and a similar level of larval survivorship between Cry3Bb1 and non-Bt maize (Fig 2 and Table 2). Farmers planted continuous maize fields to Cry3Bb1 maize for a similar number of years as current problem fields, but they used significantly more soil-applied insecticide [40]. Dunbar et al. (2016) reported the following percentages for annual use of soil insecticides at planting in maize fields (Mean ± SE): current problem fields (0 ± 0%), past problem fields (14 ± 5%), rotated maize fields (2 ± 2%), and continuous maize fields (31 ± 10%). These percentages were based on the number of years, between 2003 and the year the field was sampled (either 2013 or 2014), in which soil insecticide was applied at planting. Application of soil insecticide reduces feeding injury to maize roots from corn rootworm larvae, but may have only a minimal effect on emergence of adults (Petzold-Maxwell et al. 2013). This is because insecticides tend to kill larvae mainly in a narrow zone in the soil around the base of the maize plant (Petzold-Maxwell et al. 2013). In the case of Bt maize, soil insecticide is expected to reduce root injury but not substantially reduce survival to adulthood for Bt-resistant individuals. Thus, the rate of resistance evolution should be similar in Bt maize fields with versus without the application of soil-applied insecticide. Because continuous maize fields and rotated maize fields were selected based solely on cropping history, we expect the rates of insecticide use to be representative of the regions of the state (northern half) where the vast majority of the fields occurred. Our results suggest that the use of soil insecticide might have prevented continuous maize fields from becoming problem fields (i.e., suffering > 1 node of root injury to Cry3Bb1 maize) by protecting roots, but it did little to delay the evolution of Cry3Bb1 resistance. Shrestha et al. [49] found that the combination of soil insecticide and Cry3Bb1 maize protected the maize roots against Cry3Bb1-resistant populations of western corn rootworm, but it did not reduce adult emergence for western corn rootworm. The results of this study further support the conclusion of past work, which found that the use of soil-applied insecticide with Bt maize was not an effective strategy for delaying Bt resistance by western corn rootworm [55].

Where we were able to evaluate Cry3Bb1 resistance with both diet-based and plant-based bioassays (Tables 3 and 4; Fig 3), we found a significant positive correlation between. Additionally, LC_50_ values for Cry3Bb1-susceptible control populations were similar to past research [44, 56] as were levels of survival on Cry3Bb1 maize in plant-based bioassays [13, 17, 18]. This suggests that both plant-based and diet-based bioassays can be useful for quantifying Bt resistance.

Both diet-based and plant-based bioassays showed that some heterogeneity existed within the landscape for the level of adaption to Cry3Bb1 maize, although evidence of resistance was found for all field populations. Among individual fields, some significant differences in LC_50_ values were detected and for some individual populations an effect of Bt maize was found for either survival or development (Tables 3 and 4). Because of the limited dispersal of western corn rootworm [25], it is likely that resistance will evolve geographic mosaic, evolving more quickly in some areas than others as a result of greater localized selection intensity and reduced migration of non-adapted individuals from neighboring fields [57]. Such spatial heterogeneity in insect adaptation is often observed in agricultural systems, such as the recent adaptation of fall armyworm populations to Bt maize in Brazil [58] or the case of adaptation to host-plant resistance in wheat by Hessian fly [59]. To the extent that resistance is controlled by fine-scale localized factors it may be possible for the actions of an individual farmer to delay resistance within their field and consequently preserve the efficacy of a pest-management tool such as Bt maize.

In summary, Cry3Bb1 resistance was widespread and found in all types of maize fields sampled in Iowa as part of this study. Currently available management options including Cry34/35 maize (either as a single gene or a pyramided) and crop rotation appear to be useful in reducing the level of Cry3Bb1 resistance in the landscape. However, it is unclear whether or not this reduction in resistance has practical importance in terms of reducing root injury to Cry3Bb1 maize or preserving yield. Dunbar et al. [40] found that the abundance of western corn rootworm was significantly lower in rotated maize fields, continuous maize fields and past problem fields when compared to current problem fields. This indicates that effects of Cry3Bb1 resistance can still be mitigated, with farmers possessing the tools necessary to reduce populations of this pest. However, instances of Cry3Bb1 resistance also illustrate the potential for western corn rootworm to adapt to Bt maize and highlight the need for diversified management approaches to achieve long-term, sustainable management of this pest.
